# Positioning patients to partner: exploring ways to better integrate patient involvement in the learning health systems

**DOI:** 10.1186/s40900-023-00459-w

**Published:** 2023-07-10

**Authors:** Nakia K. Lee-Foon, Maureen Smith, Sarah M. Greene, Kerry Kuluski, Robert J. Reid

**Affiliations:** 1 Institute for Better Health, Trillium Health Partners, 100 Queensway West, Clinical and Administrative Building, 6th Floor, Mississauga, ON L5B 1B8 Canada; 2 Ottawa, Canada; 3 SG Strategies, 1249 NE 89th Street, Seattle, WA 98115 USA; 4 Institute of Health Policy, Management and Evaluation, University of Toronto, 155 College St 4th Floor, Toronto, ON M5T 3M6 Canada

**Keywords:** Learning health system, Patient engagement, Stakeholder engagement, Community based, Patient involvement, Research communities of practice, Patient partners

## Abstract

Globally, health systems are increasingly striving to deliver evidence based care that improves patients’, caregivers’ and communities’ health outcomes. To deliver this care, more systems are engaging these groups to help inform healthcare service design and delivery. Their lived experiences—experiences accessing and/or supporting someone who accesses healthcare services—are now viewed by many systems as expertise and an important part of understanding and improving care quality. Patients’, caregivers’ and communities’ participation in health systems can range from healthcare organizational design to being members of research teams. Unfortunately, this involvement greatly varies and these groups are often sidelined to the start of research projects, with little to no role in later project stages. Additionally, some systems may forgo direct engagement, focusing solely on patient data collection and analysis. Given the benefits of active patient, caregiver and community participation in health systems on patient health outcomes, systems have begun identifying different approaches to studying and applying findings of patient, caregiver and community informed care initiatives in a rapid and consistent fashion. The learning health system (LHS) is one approach that can foster deeper and continuous engagement of these groups in health systems change. This approach embeds research into health systems, continuously learning from data and translating findings into healthcare practices in real time. Here, ongoing patient, caregiver and community involvement is considered vital for a well functioning LHS. Despite their importance, great variability exists as to what their involvement means in practice. This commentary examines the current state of patient, caregiver and community participation in the LHS. In particular, gaps in and need for resources to support their knowledge of the LHS are discussed. We conclude by recommending several factors health systems must consider in order to increase participation in their LHS. Systems must: (1) assess patients’, caregivers and community understanding of how their feedback are used in the LHS and how collected data are used to inform patient care; (2) review the level and extent of these groups’ participation in health system improvement activities; and (3) examine whether health systems have the workforce, capacity and infrastructure to nurture continuous and impactful engagement.

## Abstract (plain language summary)

Patients, caregivers and communities have started taking more hands on roles in health systems, partnering with healthcare providers and researchers to impact the ways healthcare services are made and delivered. Their input has been shown to improve patient health. While many systems are working to include patients, caregivers and communities in helping improve healthcare, this work often focuses on collecting and analyzing patient data without using it in a timely way. Also, the level of their input can vary and is often limited to the start of a research project. As more health systems recognize the importance of their input in creating better healthcare, some are using different approaches to make this feedback a constant part of their systems. The learning health system (LHS) is one approach that can support deeper and ongoing patient, caregiver and community involvement in health system change. In the LHS, projects are frequently reviewed and feedback used to help health systems make changes as they go. While their involvement is critical to a well functioning LHS, it is unclear what this involvement looks like. This commentary reviews the current state of this involvement. We offer readers a way forward and suggestions to help them determine if they are actively including patients, caregivers and communities in their LHS. Suggestions include reviewing: (1) the ways data are collected and used; (2) how patients, caregivers and communities are involved in health system improvement efforts; and (3) whether or not systems have the tools needed to frequently partner with these groups.

## Background

I see patients’, caregivers’ and citizens’ roles in the Learning Health System as one leg of a wobbly stool. There is no doubt that the lived experiences of patients, caregivers, and citizens are a pillar of the Learning Health System. We often hear the term “embedded in the system.” That needs to be explained to us in terms that are understandable to us so that we can see concrete examples of how it is operationalized throughout the process and provide further input in how we can move forward and provide our input on the process. –Maureen Smith, Patient Partner

Globally, health systems are increasingly striving to deliver evidence based care that improves patients’, caregivers’ and communities’ (PCC) health outcomes [1]. Over the past few years, PCCs have taken more active roles in health systems, partnering with clinicians (e.g. doctors, nurses, social workers) and researchers to shape the design and delivery of healthcare services [2, 3]. The PCC’s lived experiences—individuals’ experiences accessing and/or supporting someone who accesses healthcare services—are seen by many health systems as an important part of understanding and improving care quality [3–6]. These experiences can be used to support healthcare improvements [7].

Across health systems, PCC’s involvement and impact can take many forms. They can be active members of and share decision making with their care teams. The PCCs’ may participate in healthcare organizational design and governance and research teams who conduct patient partnered research. They may also be part of patient and family advisory councils where, for example, they support the creation of quality improvement initiatives and staff hiring and training [7]. The PCC’s input in designing services greatly increases the chance that services reflect the values and meanings of its current and future users [8, 9]. Additionally, their involvement can lead to improved policy making along with better patient health outcomes [2]. Despite their positive impact on health systems, the level of PCCs’ involvement in and the incorporation of their perspectives into healthcare redevelopment can fluctuate or be limited in scope [3, 6]. For instance, PCC’s involvement often occurs at the beginning of a project and then gradually subsides [6, 10]. They tend to have less of a role in the later stages of a project such as helping inform the uptake and dissemination of a healthcare innovation [6].

Many health systems state that they work to involve PCC in improving healthcare [11]. However, this ‘work’ often focuses on patient data collection and analysis and quality improvement measures without sufficiently acting on the analyzed findings in a meaningful and rapid way [12]. This slow pace may be due to the challenges of change management and evidence mobilization in healthcare, as well as competing clinical priorities [13]. Additionally, grant funding is often time limited, preventing sustainable PCC involvement. As more institutions recognize that most of what influences patients’ health and wellbeing (e.g. social determinants of health) occurs outside their clinical walls, some have begun implementing different approaches to studying and applying the findings of PCC informed care initiatives in a timely fashion [10].

The learning health system (LHS) is one such framework that can support deeper and ongoing PCC engagement in healthcare change initiatives. The framework calls for continuous PCC involvement across all stages of healthcare initiatives’ development. Their involvement is considered vital to the conceptualization and successful operationalization of initiatives [1]. The PCC’s involvement is seen as the impetus for healthcare change [14]. The LHS helps health systems marry research with quality improvement through a continuous cycle of data collection and analysis. Pre-existing clinical and sociodemographic data from sources such as electronic health records and diverse patients, programs and healthcare settings are used. This allows the LHS framework to bypass the commonly used approach for generating healthcare data in a research context and avoid delays typical of a protracted research process [15]. Analyzed data are then given back to healthcare providers and decision makers (e.g., healthcare leaders, policy makers), adjustments made using this “real-time feedback” and the healthcare improvement cycle is continued [15, 16].

Considering that many health systems centre their work on PCC healthcare and the LHS framework emphasizes PCC involvement, the LHS is well positioned to ensure that patient voices continuously inform research and, in turn, practice in real-time. However, great variability exists as to what their involvement in the LHS means in practice [12]. As such, this commentary delves into the current state of PCC involvement in the LHS and ways to ensure PCCs are actively involved along every step of the LHS. The commentary is informed by the literature and experiences of an academic-community collaborative comprised of a patient partner and public health, community based research and patient and caregiver scholars.

## Patients as more than data donors

Over a decade after patients were identified as critical for catalyzing change in the LHS [17], much of the recently published LHS literature have focused on generating and translating knowledge and enhancing patient care through data [16]. There continues to be a gap in understanding patients’ roles in LHS beyond being ‘donors’ of data. Quality performance measures and healthcare outcome data continue to supplant direct PCC feedback. As noted by Kuluski and Guilcher [16], measurement tools often do not provide researchers the opportunity to understand PCCs, their abilities and the social contexts (e.g. knowledge of caregivers’ needs) they experience. Literature exploring PCCs’ perspectives and understanding of their specific role in LHS is sparse. Research examining the impact of varying levels of PCC involvement on LHS healthcare outcomes and quality improvement are lacking.

When PCCs are actively involved in applied health research and quality improvement [12], tensions can often arise when research significance, communities’ and health systems’ key issues and capabilities are misaligned [18]. This may be prompted by the continued, traditional healthcare research funding approach where proposals are selected based on their scientific significance, funding agency priorities, investigator knowledge and the potential scientific impact of newly created, generalizable information. This approach counters local systems and community prioritized research that can be multi-faceted, narrower in scope [18] and may have not yet garnered sufficient attention from academia to foster its own body of peer reviewed literature. The resulting production and use of evidence from this traditional research approach may not actively include PCC.

## Lack of guidance to support PCC’s understanding of the LHS

For many, the LHS remains an abstract concept and, for some, a buzzword. Although literature on frameworks, and examples of LHS-informed interventions exist [12], articles focused on how patients learn about and understand LHS are lacking. This is a curious oversight, given that LHS literature underscores the need for PCC engagement and their active participation in shaping healthcare [12, 16]. A scoping review of LHS articles from 2016 to 2020 found articles discussing the level of patient involvement in LHS were scarce [12]. As such, it should come as no surprise that anecdotal evidence indicates many patients are unfamiliar with the LHS, an unfamiliarity that results from a variety of factors. For instance, many scholars may be more focused on developing and revising LHS frameworks than on examining LHS’s impact on various healthcare issues and PCCs’ understanding of LHS. Furthermore, many funders have not emphasized the development of methods, language and approaches to help integrate PCCs into the LHS.

The literature provides limited insight on the role clinicians and healthcare institutions play in shaping PCCs’ understanding of and ways they can contribute to the LHS. As the LHS is a merging of healthcare delivery and research systems, institutions–particularly those without an embedded research unit or team–may find it difficult to determine best practices for PCCs’ involvement. This is unfortunate as opportunities for synergies and mutual understanding between PCCs involved in research and quality improvement initiatives exist. Further, low health literacy—the limited ability to access, comprehend, process and apply health information—can act as a barrier to participating in health system improvement for some PCCs [19]. Finally, healthcare institutions may be failing to engage PCCs as collaborators in advancing high value healthcare delivery that best fits patients’ and caregivers’ needs [14].

## PCCs’ limited involvement in the LHS

Despite health systems’ increased interest in LHS, PCCs are limited in their involvement in the LHS. No common language, tools or frameworks for discussing and operationalizing LHS exist, making it likely that many healthcare institutions are using this approach without explicitly naming it as such [12]. Tools that exist often focus on the ‘average patient’ failing to engage and reflect diverse voices and needs, particularly those from equity deserving groups who are marginalized due to their socio-economic status, gender identity, racialization, sexual orientation and other categories of difference. Limited literature discusses the creation of a practical, equitable LHS framework co-designed by PCCs [12]. The lack of commonly used language and LHS frameworks makes it difficult to explain the benefits of obtaining care in and the importance of their role in shaping the LHS. Furthermore, there is a lack of research focused on understanding PCCs’ experience and perspective of LHS. Many PCCs remain unaware of how their involvement in LHS informs healthcare practice. At the time of writing, courses dedicated to teaching the LHS approach and methods are minimal, making it difficult for some health system leaders to understand how best to introduce and teach various components of the LHS to PCCs.

## Missed opportunities for PCC involvement in the LHS

PCCs’ engagement in health systems may stem from a diagnosis or condition and their perceived ability to trigger change. This engagement can occur via roles in different spheres like patient-partnered research and patient and family advisory councils. Although synergies exist between these two roles, PCCs may not interact and differ in their approach to supporting healthcare issues and challenges. It may be difficult for them to distinguish between LHS informed versus non-informed healthcare institutions and no specific guidelines exist on how best to integrate PCCs into LHS [10]. This gap is a missed opportunity for health systems to learn directly from PCCs how to increase patient satisfaction, healthcare service delivery and, ultimately, health outcomes. As PCC involvement is vital to moving the gears of LHS along, fostering PCC’s awareness and understanding of this concept and how and where it is operationalized to include their lived experiences and insights is vital.

## A way forward

Despite the aforesaid gaps in PCC involvement in the LHS, we believe there are several ways to begin mending these gaps. When seeking to involve patients in LHS, several key items must be considered. Health systems must first ask themselves: “exactly what is being done with the data we collect and how can we use it to fulfill our mission of caring for patients?” This question is vital to enhancing patient centered-care strategies at the local/organizational level as systems are prompted to reflect on why data are being collected and how the findings will inform care. This reflection is lacking in many systems. In order to maintain PCC’s trust of health systems, systems that have historically failed equity deserving groups [13], PCCs must be shown how their experiences and input are used in LHS. Additionally, they must be provided with accessible information and training to help them better participate and co-determine their role and impact in LHS.

Second, healthcare institutions must ask themselves: “how are patients and caregivers involved in the learning activities (e.g., research prioritization, evidence collection, data synthesis, dissemination) of the health system’s planning, improvement and knowledge dissemination efforts, and at what level (e.g., consultations, involvement, collaboration, lead/support)?” As previously noted, PCCs are often not included in every stage of health system work and only consulted after data analysis or implementation activities occur. They are not told how analyzed data and research fit into the care delivery and improvement approach, nor how it will be used to inform healthcare changes and future research. True LHS informed patient-centered care approaches require PCCs to actively participate at every step of the research process from research design to then applying study findings to patient-centered care initiatives. They must be made aware of their work’s impact on healthcare systems and whether these systems are supporting their needs. Given the dearth of research on PCC’s engagement in LHS, case studies, learning communities, or other exemplars that show effective PCC engagement in learning activities are crucial. At the same time, health systems must examine whether they have the capacity to build the core values and infrastructure that foster continuing and substantive PCC engagement and whether its workforce have the skills needed to collaborate with PCCs to develop more effective healthcare [20]. This examination and follow-up aligns with LHS and ensures that care truly reflects patient and caregiver needs.

Finally, the LHS offers a tremendous opportunity to dismantle the silos of patient-partnered research and healthcare quality improvement research, both of which are vital components of a successful LHS. Bringing these groups together would enhance their respective strengths, accelerate the pace of care improvement, and likely enhance the types of evidence that can be shared and meaningfully synthesized. We believe these aforesaid recommendations will give rise to healthcare institutions who truly partner with a diverse array of PCCs and ultimately care that responds to their healthcare priorities and needs.

## Data Availability

Not applicable.
